# The Hideous Side of Acute Pancreatitis: A Case of Pancreatitis-Induced Atypical Hemolytic Uremic Syndrome

**DOI:** 10.7759/cureus.87980

**Published:** 2025-07-15

**Authors:** Hasan Al-Ali, Ahmed Elmogy, Hina Arsh, Harrison Rhee

**Affiliations:** 1 School of Medicine, St. George's University School of Medicine, True Blue, GRD; 2 Cardiovascular Medicine, Cairo University, Cairo, EGY; 3 Internal Medicine, Icahn School of Medicine at Mount Sinai, Queens Hospital Center, New York City, USA

**Keywords:** atypical hemolytic-uremic syndrome (ahus), clinical suspicion, pancreatitis complication, thrombotic microangiopathy, thrombotic thrombocytopenic purpura (ttp)

## Abstract

Thrombotic microangiopathy (TMA) is a medical emergency characterized by thrombocytopenia, microangiopathic hemolytic anemia, and acute kidney injury (AKI). Its subtypes, including thrombotic thrombocytopenic purpura (TTP), atypical hemolytic uremic syndrome (aHUS), and secondary TMA, require rapid differentiation due to divergent treatments. Acute pancreatitis, in some cases, can trigger TMA through systemic inflammation and complement activation. A middle-aged male with chronic kidney disease and diabetes was admitted for acute pancreatitis, likely in the setting of glucagon-like peptide-1 (GLP-1) use. On day 3, he developed progressive thrombocytopenia, anemia, and worsening renal function. Peripheral smear showed schistocytes; laboratory values were remarkable for hemolysis. Given the high suspicion for TMA and a PLASMIC score of 5, empiric plasma exchange and corticosteroids were initiated, which showed clinical improvement. ADAMTS13 activity returned at 99%, effectively excluding TTP. Complement studies for aHUS were inconclusive and were obtained post-plasma exchange, which can alter results. The patient completed five sessions of plasma exchange and was discharged in stable condition. A follow-up was arranged for hematology. This case highlights the diagnostic complexity of TMA triggered by acute pancreatitis and the necessity of early empiric treatment. It also calls attention to the limitations of delayed ADAMTS13 and complement testing and advocates for broader implementation of rapid in-house assays to guide timely, precise therapy.

## Introduction

Thrombocytopenia with anemia and concurrent acute kidney injury (AKI) presents a clinical red flag that mandates urgent evaluation for thrombotic microangiopathy (TMA), a group of syndromes that includes thrombotic thrombocytopenic purpura (TTP) and hemolytic uremic syndrome (HUS). As per previous studies [1], the pathophysiology of each disease varies. TTP primarily results from a severe deficiency of ADAMTS13, which results in an increased number of platelet activations and thrombosis, causing thrombosis, which is likely to be neurological [2]. As per HUS, typical cases usually arise mostly after intestinal infection, and atypical cases are due to complement dysregulation. Various studies [3] further classified typical HUS as being caused only by the Shigatoxigenic *Escherichia coli* (STEC), most predominantly in children with notable antibiotic use for gastroenteritis [4]. For this case report, atypical HUS will be classified as TMA due to genetic complement mutations, such as by the CFH gene [5], and secondary TMA will cover the other factors. Regarding HUS, supportive therapy is the mainstay treatment, primarily involved in minimizing harmful intervention and monitoring for complications [4]. On the other hand, TTP and aHUS must be corrected with either plasmapheresis or complement inhibition. As referenced by other studies, TTP is primarily treated with plasmapheresis, whilst aHUS benefits more from eculizumab as a C5 inhibitor [4]. Additionally, with respect to aHUS, previous studies suggest eculizumab as superior if complement studies confirm atypical HUS, with plasmapheresis being considered if eculizumab or complement studies are not yet available [6]. Lastly, secondary TMA is managed by treatment of the underlying causative disease, with some efficacy of plasmapheresis in some studies [7].

Pancreatitis, while not traditionally considered a primary instigator of TMA, has increasingly been implicated as a potential trigger due to its ability to incite systemic inflammation, endothelial injury, and complement activation [8]. Furthermore, the fibrinolytic system is impaired due to inhibition of plasminogen activator inhibitor type 1; this system's inflammation is associated with hypercoagulability, which can lead to microvascular thrombosis and subsequent organ damage [9].

This report presents a detailed account of a patient who developed severe thrombocytopenia and AKI after an episode of acute pancreatitis. The clinical and laboratory course suggested a diagnosis of TTP or aHUS, leading to the prompt initiation of empiric therapy before confirmatory tests became available. Our case highlights the diagnostic ambiguity and urgency in managing TMA in the setting of systemic inflammation, and the potential for pancreatitis to unmask underlying microangiopathies.

## Case presentation

A middle-aged patient with a past medical history of stage 3A chronic kidney disease (CKD) and diabetes mellitus type 2 presented to the emergency department with sudden-onset, severe epigastric abdominal pain radiating to the back. The pain was accompanied by five episodes of non-bloody, non-bilious vomiting, as per the patient. The patient has a past surgical history of endoscopic retrograde cholangiopancreatography (ERCP) and laparoscopic cholecystectomy two years ago. He currently takes metformin, semaglutide, and quetiapine. Furthermore, no significant family history is noted. Lastly, no use of smoking or alcohol is reported. All laboratory results are listed in Table 1.

**Table 1:** Significant Laboratory Results Daily laboratory values during hospitalization are presented. Plasmapheresis was performed on days 6 through 10, which are indicated in bold. AST: aspartate aminotransferase; ALT: alanine aminotransferase

Laboratory Results (Reference Range)	Day of Onset											
	1	2	3	4	5	6	7	8	9	10	11	12
Hemoglobin (13.0-17.0 g/dL)	14.8	14.4	11.9	11.1	9.8	10.5	9.60	9.50	9.40	9.20	9.8	9.5
Platelets (150-450 x 10^3^/mcL)	245	152	40	21	22	54	88	101	172	198	207	219
Creatinine (0.50-1.30 mg/dL)	2.77	3.70	4.24	4.17	4.26	3.42	3.13	2.60	2.06	1.89	1.51	1.29
Lipase (13-60 U/L)	2476	-	-	-	-	-	-	-	-	-	-	-
Direct Bilirubin (0.0-0.20 mg/dL)	0.40	-	1.30	1.30	0.70	0.30	<0.08	-	0.10	0.10	0.10	0.10
Indirect Bilirubin (0.0-1.0 mg/dL)	0.40	-	1.7	1.2	0.60	0.30	-	-	0.20	<0.20	<0.20	<0.20
AST (5-40 U/L)	27	-	79	141	83	125	42	-	40	31	75	69
ALT (0-41 U/L)	19	-	22	45	43	71	37	-	33	43	108	141

**Figure 1:**
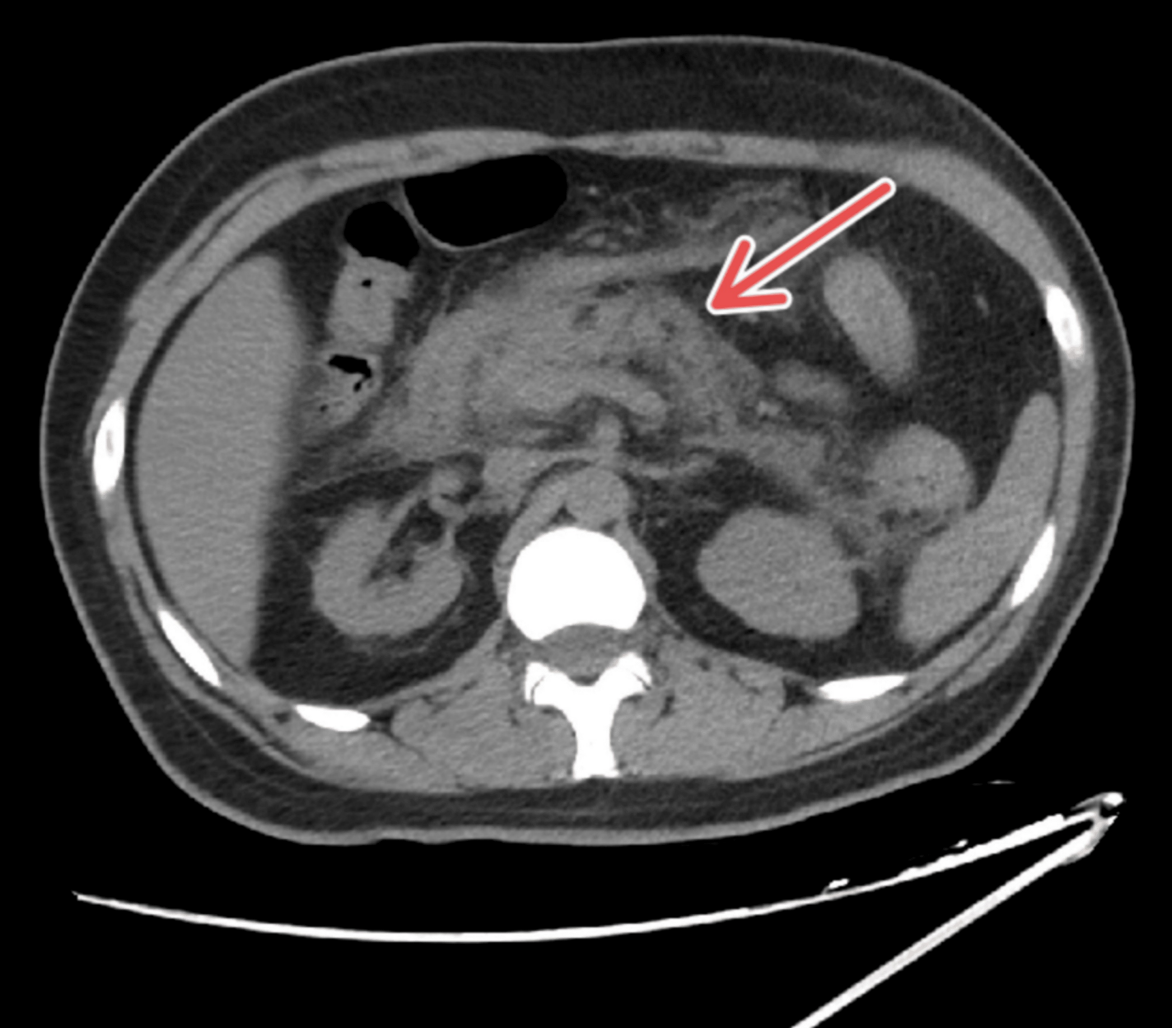
CT of the Abdomen and Pelvis Without Contrast Abdominal CT at the level of the epigastrium. The red arrow marks the peri-pancreatic stranding, compatible with the diagnosis of acute pancreatitis.

On arrival, he was tachycardic and afebrile, with remaining vitals within normal limits. Physical examination revealed normoactive bowel sounds with mild tenderness in the epigastrium without peritoneal signs. Laboratory results were notable for a lipase level of 2476 U/L. Liver function tests and complete blood counts were within range. Creatinine has been elevated at 2.77 mg/dL from a baseline of 1.5-2.0 mg/dL since 2022. CT imaging was obtained without contrast due to the patient's history of CKD, which showed peri-pancreatic stranding, without necrosis, pseudocyst, or common bile duct dilatation as displayed in Figure 1. The right upper quadrant ultrasound was inconclusive. The etiology was presumed to be glucagon-like peptide-1 (GLP-1)-related. Furthermore, glucose on admission was 119 with an anion gap of 12, ruling out diabetic ketoacidosis. The patient was admitted for acute pancreatitis and started on fluid resuscitation with lactated Ringer's solution, advanced diet as tolerated, and appropriate pain control. Additionally, based on our suspicion of etiology, semaglutide was stopped due to its risk of inducing pancreatitis.

On hospital day 2, the patient developed a fever of 102.4°F, a rise in liver function tests with aspartate aminotransferase (AST) reaching 72 U/L, and rising creatinine (3.7 mg/dL). Blood cultures were obtained. ERCP was inconclusive, and the patient remained under supervision. By the third day, abdominal pain was reduced, and the patient started tolerating oral intake; however, his laboratory results were abnormal. He started developing thrombocytopenia, which was from a baseline of 245,000 to 40,000 platelets per microliter. Notably, on the same day, he had a hemoglobin level of 11.9 g/dL, falling 20.7% from his baseline. This prompted a workup initially to confirm the patient's thrombocytopenia, in which a blood smear showed no platelet clumping and the presence of moderate anisocytosis and poikilocytosis. Additionally, a significantly high number of schistocytes were observed. At this time, the patient started tolerating oral intake and reported improvement of his abdominal pain, and his pancreatitis symptoms had been resolved clinically, which brought suspicions of a new underlying mechanism causing these abnormalities. His creatine continued to rise to 4.24 mg/dL with elevated bilirubin levels (total bilirubin increased from 1.00 mg/dL to 3.00 mg/dL, and indirect from 0.40 mg/dL to 1.30 mg/dL).

Over the next few days, the patient remained febrile and platelets dropped until the evening of the fourth day, when serial complete blood counts showed a platelet decrease from 40,000 to 21,000 platelets per microliter. Blood cultures were negative for any organisms. The workup for disseminated intravascular coagulation (DIC), including fibrinogen and D-dimer levels, was unremarkable. Hemolysis studies were ordered, which highlighted a lactate dehydrogenase (LDH) level of 1347 U/L, a haptoglobin level of less than 20 g/L, and indirect bilirubin remained elevated at 1.20 mg/dL. A hematology consultation suggested a low possibility of TMA; therefore, additional monitoring of laboratory results was required. The following day, platelets remained low at around 20,000 platelets per microliter, with hemoglobin reducing to 9.8 g/dL (34.7% decrease), and creatinine elevated at 4.26 mg/dL. This fulfilled the triad of hemolysis, AKI, and thrombocytopenia, leading to hematology, suggesting starting plasma exchange therapy due to the risk of TTP. A PLASMIC score, which is a validated clinical prediction tool used to rapidly estimate the probability of ADAMTS13 deficiency in adults presenting with suspected TTP, was used to evaluate the next step of management. The score was a 5, which indicated an intermediate risk for TTP; therefore, plasmapheresis was indicated. The patient received folate due to hemolysis and was started on 70 mg of prednisone daily (calculated from 1 mg/kg). He was then transferred to the stepdown unit with routine tests. Additionally, the ADAMTS13 test was ordered before the initiation of plasma exchange.

Five daily sessions of plasma exchange were ordered, and the patient's status was monitored daily. After the first exchange, the patient tolerated it well, and platelets were elevated to 54,000 platelets per microliter. The second exchange had similar results, which revealed a platelet count of 88,000 platelets per microliter. Hemoglobin levels were stable, and creatinine slowly corrected back to 2.6 mg/dL. Additionally, LDH levels trended down throughout treatment, with a level of 439 U/L after the second session, a drop from 1347 U/L before plasma exchange. ADAMTS13 came out after two days of plasma exchange, yielding a 99% activity rate, which indicates a very low likelihood of TTP. At that time, a workup for aHUS was ordered and sent for testing. The patient also tested positive for rhinovirus, and immune thrombocytopenic purpura (ITP) was initially suspected but ruled out due to AKI and hemolysis. Plasma exchange was continued for three more sessions, and platelets at the end showed a value of 188,000 platelets per microliter. His LDH at the end of the plasma exchange was 205 U/L, which was within the reference range (135-225 U/L). He is currently stable with no complaints, and laboratory results are within his baseline. The presumed diagnosis was initially pancreatitis-induced TTP; however, aHUS is more likely considering the ADAMTS13 level. Treatment of aHUS is ideally eculizumab; however, as previously stated, due to no unequivocal evidence of aHUS diagnosis, plasma exchange was commenced instead and yielded positive findings. Workup of aHUS was released later and showed normal complement levels and activity, which reduces the likelihood of aHUS, but it is to be noted that this workup was obtained on the second day of plasmapheresis, which can lead to invalid results due to correction by plasma exchange. Additionally, it was observed that earlier management was more important than withholding treatment, since the management was similar. Secondary TMA would have been considered but ruled out due to observed clinical improvement of pancreatitis ahead of thrombocytopenia, which does not follow the pattern of platelet resolution with underlying disease treatment (in this case, pancreatitis).

Given the patient's response to plasma exchange and steroids, no eculizumab was initiated during the hospitalization. The patient was discharged on hospital day 12 with outpatient follow-up in hematology and nephrology. Plans included repeat complement and genetic studies to further delineate the etiology.

## Discussion

This case illustrates the diagnostic complexity and clinical urgency of TMA in the setting of acute pancreatitis. The triad of thrombocytopenia, microangiopathic hemolytic anemia, and AKI is highly suggestive of a TMA spectrum disorder, necessitating immediate intervention due to the complications of TTP and aHUS without prompt therapy.

The diagnostic dilemma initially centered around differentiating between TTP, aHUS, and secondary TMA. Given the patient’s rapid platelet decline, hemolysis, and worsening renal function despite clinical resolution of pancreatitis, primary TMA entities like TTP and aHUS were high on the differential. Although acute pancreatitis has been associated with secondary TMA, such cases typically exhibit resolution of hematologic abnormalities in parallel with control of the underlying disease [8]. In contrast, our patient’s thrombocytopenia and hemolysis developed after improvement in pancreatic inflammation, undermining the diagnosis of secondary TMA.

Empiric plasma exchange was promptly initiated given the life-threatening potential of TTP and the PLASMIC score of 5, which reflects an intermediate pretest probability [10]. The rapid hematologic response to plasma exchange further supported a primary TMA process. However, the patient’s ADAMTS13 activity of 99% effectively ruled out TTP as the etiology [11]. While aHUS became more probable at this stage, interpretation of complement studies was confounded by prior plasma exchange, which can transiently affect complement markers and obscure an accurate diagnosis [12].

The main issue that was faced during this diagnosis for TTP was the duration it took for testing, which halted diagnosis confirmation and resulted in the trial of plasmapheresis as empiric testing, when other, more reliable treatments could have been applied. Traditional reference assays, such as enzyme-linked immunosorbent assay (ELISA) or fluorescence resonance energy transfer-substrate von Willebrand factor 73 (FRETS-VWF73), typically require several hours to perform and, when sent to external reference laboratories, results are often available only after several days [13-14]. This delay necessitates empiric treatment decisions in many clinical settings. However, new advancements of ADAMTS13 testing have emerged rapid, fully automated chemiluminescent immunoassays (CLIA), such as the HemosIL AcuStar, which can provide ADAMTS13 activity results within approximately 30-35 minutes of sample processing. These rapid assays are increasingly available in tertiary care centers and have demonstrated high sensitivity and specificity for severe ADAMTS13 deficiency. In summary, results can be available in as little as 30-35 minutes with rapid in-house assays, but may take several days if testing is sent to a reference laboratory [15].

## Conclusions

This case reviews the diagnostic and therapeutic challenges posed by TMA in the context of acute pancreatitis. Despite initial suspicion for TTP, a high ADAMTS13 activity effectively excluded the diagnosis. Although aHUS remained a consideration, interpretation of complement studies was confounded by prior plasma exchange. Nonetheless, the patient exhibited a robust clinical and hematologic response to empiric plasmapheresis and corticosteroids, supporting the presence of a primary TMA. This case highlights the importance of early recognition and treatment initiation in suspected TMA, particularly when diagnostic clarity is delayed. It also emphasizes the need for wider availability and use of rapid ADAMTS13 assays to expedite diagnosis and optimize care.
